# Supplementary material for: Betaine Alleviates Bisphosphonate-Related Osteonecrosis of the Jaw by Rescuing BMSCs Function in an m6A-METTL3-Dependent Manner
Source: Int J Mol Sci. 2025 May 29;26(11):5233. doi: 10.3390/ijms26115233 (PMC12154196; doi:10.3390/ijms26115233)
Supplement: Supplementary file 1 [file ijms-26-05233-s001.zip › ijms-3620730-supplementary Table S1.pdf]

Supplementary Table S1. Primers used in RT-PCR

Genes	Primers 5'- 3'
GAPDH	F: TCGGAGTCAACGGATTTGGT
	R: TTCCCGTTCTCAGCCTTGAC
METTL3	F: AGATGGGGTAGAAAGCCTCCT
	R: TGGTCAGCATAGGTTACAAGAGT
